# Creative challenge: Regular exercising moderates the association between task-related heart rate variability changes and individual differences in originality

**DOI:** 10.1371/journal.pone.0220205

**Published:** 2019-07-22

**Authors:** Christian Rominger, Ilona Papousek, Andreas Fink, Corinna M. Perchtold, Helmut K. Lackner, Elisabeth M. Weiss, Andreas R. Schwerdtfeger

**Affiliations:** 1 Department of Psychology, University of Graz, Graz, Austria; 2 Otto Loewi Research Center, Section of Physiology, Medical University of Graz, Graz, Austria; University of Brasilia, BRAZIL

## Abstract

Coping with mental challenges is vital to everyday functioning. In accordance with prominent theories, the adaptive and flexible adjustment of the organism to daily demands is well expressed in task-related changes of cardiac vagal control. While many mental challenges are associated with increased effort and associated decreased task-related heart rate variability (HRV), some cognitive challenges go along with HRV increases. Especially creativity represents a cognitive process, which not only results from mental effort but also from spontaneous modes of thinking. Critically, creativity and HRV are associated with regular exercising and fitness. Furthermore, the cross-stressor adaptation theory suggests that changes in cardiac reactions to physical challenges may generalize to mental challenges. In line with this idea the amount of regular exercising was hypothesized to moderate the association between HRV changes and creativity. A sample of 97 participants was investigated. They reported the amount of regular exercise and their ECG was measured at baseline and during a creativity task. An association between task-related HRV changes and originality as a function of participants’ amount of regular exercise was found. Participants reporting more regular exercising produced more original ideas when they had higher HRV increases during the task, while more sedentary participants showed the opposite association. Results suggest that individuals with a higher amount of regular exercise achieve higher originality probably via the engagement in more spontaneous modes of thinking, while more sedentary people may primarily benefit from increased mental effort. This supports the conclusion that higher creativity can be achieved by different strategies.

## Introduction

Diverse mental challenges are a major part of daily life, and the flexible adjustment to these day-to-day demands seems mandatory for successful coping [1]. However, this adaptive ability of self-regulation greatly varies between people and situations. Notably, mental effort, stress, and self-regulation are indexed by functions of the vagus nerve as the primary part of the parasympathetic nervous system [1–5]. A putative physiological marker of vagal control is the beat to beat variation in heart rate, the so-called heart rate variability (HRV) [6,7]. The vagal influence on HRV can be quantified in the time domain by means of RMSSD (root mean square of the successive difference in normal sinus beat intervals) and pNN50 (percentage of adjacent normal sinus beat intervals that differ by more than 50 ms) [8–10].

While the tonic HRV level potentially indicates an inter-individual trait like capacity to adapt to environmental challenges by parasympathetic/vagal control [11–13] (for a meta-analysis see [14]), task-related HRV changes represent the dynamic task-specific vagal control of the heart (i.e., vagal break) [2,15]. Therefore, task-related HRV is an even more sensitive variable in the context of regulatory efforts of the organism and the actual flexible adaption to physical and mental challenges [2,3,6,7,13,16,17]. Following from this, the adjustment of the organism to task-related challenges—e.g., self-regulatory efforts [4,13]—may be best represented by the flexible change of the HRV from a baseline to a period of mental effort.

However, not every mental challenge requires the same amount of cognitive and regulatory capacity [18,19]. Of note, while many mental tasks are associated with task-related HRV decreases [16,20,21], there is evidence that some cognitive tasks are associated with task-related increases [3,4,15]. For instance, Butler et al. [13] found HRV increases during the reappraisal of negative events [22,23], and Silvia et al. [24] reported a slight rise of vagal control during a creative ideation task. This is remarkable, since creative ideation and cognitive reappraisal (the latter as an instance of “creativity in an affective context” [25]) represent complex self-regulatory cognitive processes (e.g., flexible switch between associative and executive functions), which share important basic cognitive functions [25–28].

A unique characteristic of creative and divergent thinking tasks in contrast to traditional convergent cognitive tasks is the interplay of associative/spontaneous modes of thinking and executive top-down control processes [29–34]. Following dual process theories, creative ideation is not solely based on mental effort, which is associated with HRV decrease [24,35–38], but also on spontaneous, automatic, associative, and relaxed modes of thinking, which are presumably associated with HRV increase [39–42]. Furthermore, the flexible shift between these different modes of thinking is important for creative ideation performance [43–45]. The contribution of both modes of thinking to creative ideation may explain contradictory findings in literature. Loudon and Deininger [35] reported a negative and Bowers and Keeling [40] a positive association between indices of creative performance and HRV. Silvia et al. [24] found no significant link between task-related vagal control and originality.

However, a further reason for inconsistent findings in this context might be that cognitive performance and HRV are associated with the amount of regular physical exercise, physical fitness, and general health of people [6,8,46] (for cognition see [47,48]; but see [49]; for cognition and HRV see [50]). Furthermore, in a recent study, Latorre Román et al. [51] found an association between measures of physical fitness and creativity (see also [52,53]; similar findings for acute physical activity see, [54–56]). The cross-stressor adaptation theory [57] assumes that salutary cardiovascular reactivity and recovery due to physical training generalize from ergogenic challenges to psychogenic challenges. This generalization may lead to a differentiation of cardiac reactivity to psychological stressors between people doing more and people doing less exercise and people who might therefore be more or less physically fit [46,58–60].

Following this idea, the present study examined whether regular exercising may moderate the association between task-related HRV changes and the performance outcome in a creative ideation task. People regularly exercising more are supposed to be physically best prepared to show adaptive task-related changes of HRV [6], in order to flexibly match the adaptive states important for optimum performance outcome [1]. More precisely, it was assumed that participants with a higher amount of regular exercise may more flexibly adjust to the creative challenge and more easily switch between mental effort [24,35] and spontaneous and automatic modes of thinking [40–42]. Therefore, it was hypothesized that participants exercising more often will show higher task-related HRV during creative ideation in contrast to participants exercising less, and that in exercisers (but not necessarily in sedentary people) the HRV response will be related to task performance.

## Methods

### Participants

An a-priori power analysis was conducted using the software G*Power 3.1 [61]. The analysis indicated that a sample size of 99 participants is required to detect a medium effect (*f*^*2*^ = .15) with a power of .80 and an alpha error of 5%. One hundred and two people were recruited to participated in the study because of potential technical failures and dropout from the study. Due to recording problems of the electrocardiogram two participants were excluded. Two further participants did not comply with the instructions, and another participant was excluded because of excessive artifacts in the recorded ECG. The final sample consisted of 97 participants (54 women) with an age range from 18 to 33 years (*M* = 23.07 years; *SD* = 3.48 years). Participants’ age range was restricted to control for potentially confounding influences on HRV [15].

People with a history of major psychiatric disorders according to the Structured Clinical Interview for DSM-IV Axis I Disorders (SCID) and people, who reported having a neurological disease or using psychoactive medication were not included in the study. All participants were right-handed (assessed by a standardized hand skill test; [62]). They were requested to refrain from alcohol intake for 12 h and from drinking coffee and other stimulating beverages for 2 h prior to their appointment, and to come to the session well rested. The study was approved by the Ethics Committee of the University of Graz, approval number GZ. 39/41/63 ex 2015/16. Written informed consent was obtained from all participants.

### Amount of regular physical exercise

The Freiburger Questionnaire on Physical Activity (FQPA) [63] is a reliable and valid self-report instrument, which covers everyday physical activities (e.g., walking to work), leisure time activities (e.g., dancing), and sports activities (e.g., swimming) [64,65]. Participants are instructed to indicate the duration for each activity in minutes per week which are converted into metabolic equivalents (MET) [66]. In order to derive a measure of physical exercise potentially resulting in higher bodily fitness [67], only the reported hours of sports activity per week (e.g., running, playing soccer, swimming, *M* = 3.41 h, *SD* = 3.68 h) were considered (exercise MET: *M* = 22.16, *SD* = 26.12) [68]. Frey et al. [63] reported a re-test reliability of *r* = .98 (two weeks) for the amount of exercise per week.

### Creative thinking task and task performance

**Fig 1:**
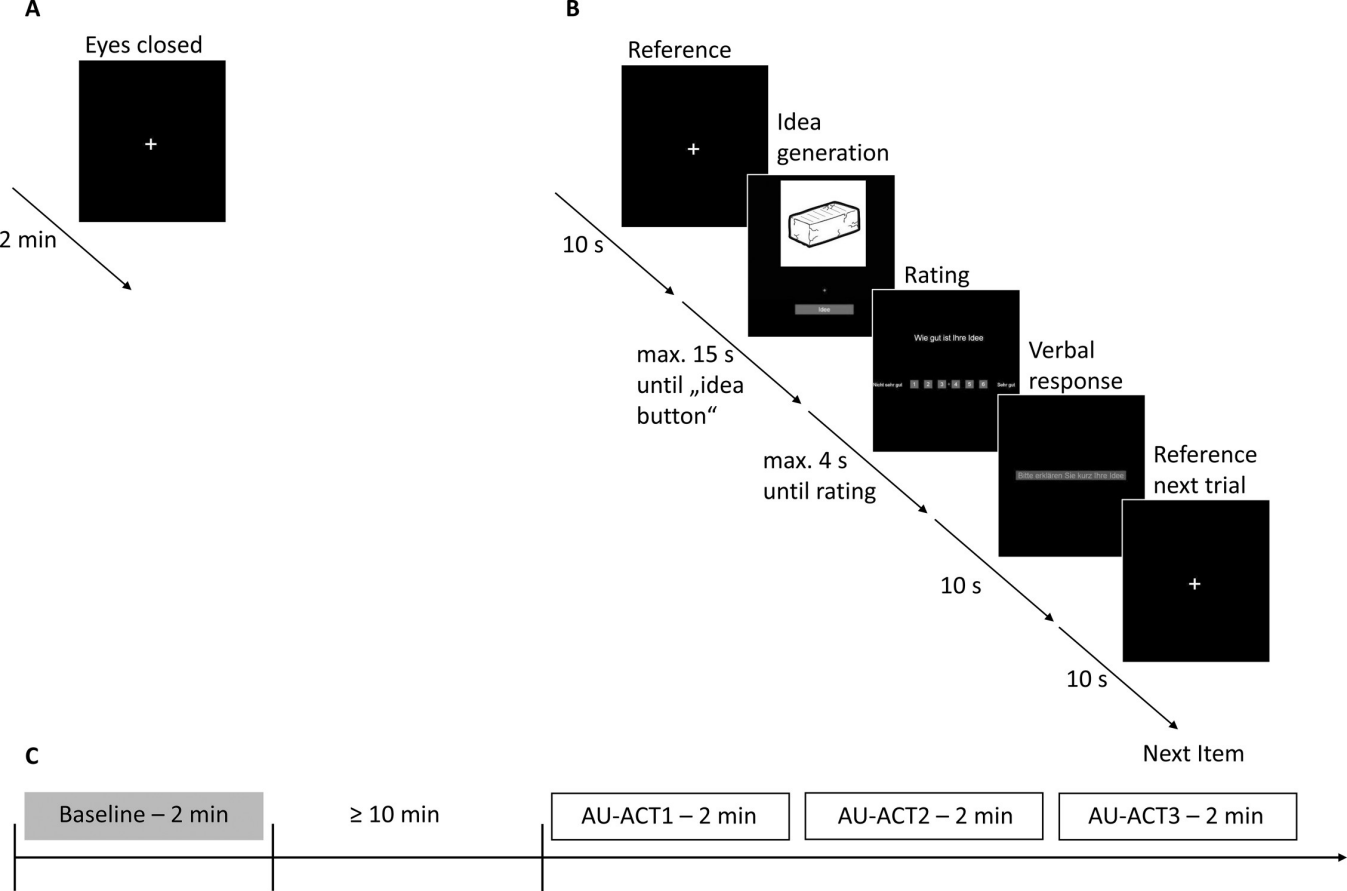
Schematic time course of the computerized (A) baseline, (B) AU task, and (C) the analysed 2 minutes time periods (Baseline, AU-ACT1, AU-ACT2, AU-ACT3).

The Alternate Uses (AU) task [69] is a verbal creativity/divergent thinking test used in numerous scientific studies [24,30,70]. The participants’ task was to generate alternative uses of common objects (e.g., umbrella, brick, or key). For the present investigation, a single answer (i.e., best idea) and self-paced version of the AU task was used. This approach was applied in order to more strongly focus on the originality aspect of creativity [26]. The self-paced procedure appropriately captures the spontaneous nature of the creative thinking process [71–75]; however, it also implicates high demands on self-regulatory mechanisms. Each trial started with a white cross (10 s), followed by a picture of a common object (idea generation phase with a max. response time of 15 s; see Perchtold et al. [26] for a similar procedure). After the “idea button” was pressed, the participants rated the originality of their idea on a 6-point Likert-scale (max. response time of 4 s) and finally voiced the idea (10 sec). The subjective rating was included to maintain the participants’ effort to produce high quality ideas for every single item of the task. With the presentation of a new fixation cross the next trial started (see Fig 1 for an illustrative summary of the time course of the task). Sixteen objects were presented in randomized order.

The voiced ideas were transcribed and rated by three independent and trained raters on a four-point Likert scale ranging from “not original” to “very original”. This procedure is a common approach in creativity research (cf. Consensual Assessment Technique; [30,76]). The originality ratings showed acceptable interrater reliability (ICC (2, k) = .66).

### Procedure

All participants were tested individually. The recording took place in a separate and quiet room, where participants were seated in a comfortable chair. During the recordings, they were monitored via a web cam to ensure that they were following the instructions. After filling in the consent form, participants filled in the questionnaire assessing the amount of regular physical exercise. Then, the ECG was applied, and the baseline was recorded for two minutes. Participants were told to relax and keep their eyes closed until they heard a signal tone. After that, participants worked on tasks not relevant for the study question, which lasted a minimum of 10 minutes (see Fig 1). Then, the AU task followed.

### Recording and quantification of HRV

The electrocardiogram was recorded using a standard limb lead II electrode configuration with a sampling rate of 500 Hz (Brainvision Research Amplifier, Brain Products^TM^). The ECG signals were manually checked for artifacts by means of the software Kubios HRV Premium version 3.0.2. The mean heart rate of the participants at the baseline level was 68.61 bpm (*SD* = 11.11 bpm). HRV was quantified in the time domain by means of the root mean square of successive differences of the successive RR intervals. RMSSD has been shown to sensitively index vagal efference [77] especially during cognitive processes [21]. RMSSD was calculated for the two minutes baseline interval and three consecutive two minutes periods during the creative ideation task, starting from the onset of the first white cross (see Fig 1). Two minutes are considered adequate to evaluate RMSSD reliably [9,15,78]. As depicted in Fig 1, the three two minutes periods of the creative thinking task were calculated for comparability reasons with baseline ([9,15]; similar procedure see, [24,60,79]). The pooled untransformed RMSSD value of the three activation periods was *M* = 49.43 ms (*SD* = 24.57 ms) and the baseline was *M* = 51.63 ms (*SD* = 27.25 ms). The RMSSD reactivity showed high variability, with both increases and decreases (*M* = -2.20 ms, *SD* = 13.24 ms, min = -49.13 ms, max = 28.12 ms).

Furthermore, to control for respiration-induced changes in HRV, ECG derived respiration (EDR) was calculated by means of software Kubios HRV Premium version 3.0.2 for the baseline (*M* = 0.23 Hz, *SD* = 0.04 Hz) and the three consecutive two minutes activation periods, which were pooled together (*M* = 0.23 Hz, *SD* = 0.03 Hz).

On average, participants completed 11.73 (*SD* = 1.78) items during the three activation periods of overall 6 minutes (min = 6 items, max = 14 items). The range of response times from item onset to activation of the “idea button” was between 2933 ms and 12 329 ms (*M* = 6522 ms, *SD* = 2231 ms).

### Statistical analysis

Because of the skewed distribution of the RMSSD values, which was visually checked, a transformation with the natural logarithm (ln) was performed for all conducted statistical analyses [15,80–82].

Firstly, to explore the overall challenging effect of the creative thinking task, a one-way analysis of variance with the within-subjects factor TIME (Baseline, AU-ACT1, AU-ACT2, AU-ACT3) and the HRV (lnRMSSD) as the dependent variable was calculated. Due to violations of sphericity assumptions, the multivariate approach was used [83]. To illustrate the interindividual differences in HRV changes, the untransformed RMSSD values were presented.

The main research question, if regular exercising moderates the association between task-related HRV changes and creative ideation performance outcome, was evaluated using standard multiple regression analysis with the task-related HRV change score, the amount of physical exercise, and the interaction term as predictors, and task performance (originality) as the dependent variable. The task-related HRV change score was calculated by regressing the baseline lnRMSSD values on the pooled lnRMSSD values of the three two minutes activation periods (AU-ACT1, AU-ACT2, AU-ACT3) [84]. This procedure results in one residualized change index with higher scores indicating a relative increase in HRV and lower scores indicating a relative decrease in HRV during the creative ideation task. To illustrate the significant interaction effect of physical exercise and task-related HRV changes on originality, predicted originality was calculated for one standard deviation below sample mean (M—1 SD) and one standard deviation above sample mean (M + 1 SD) using standard regression analysis (similar procedure see e.g., [85,86]). To control for respiratory-induced changes in HRV, this standard multiple regression analysis was re-run with the mean EDR frequency of the three activation periods as an additional predictor.

All analyses were calculated by means of IBM SPSS Statistics 25 for windows and the significance level was set to 5%.

## Results

### Overall effect of the creative thinking task on HRV

**Fig 2:**
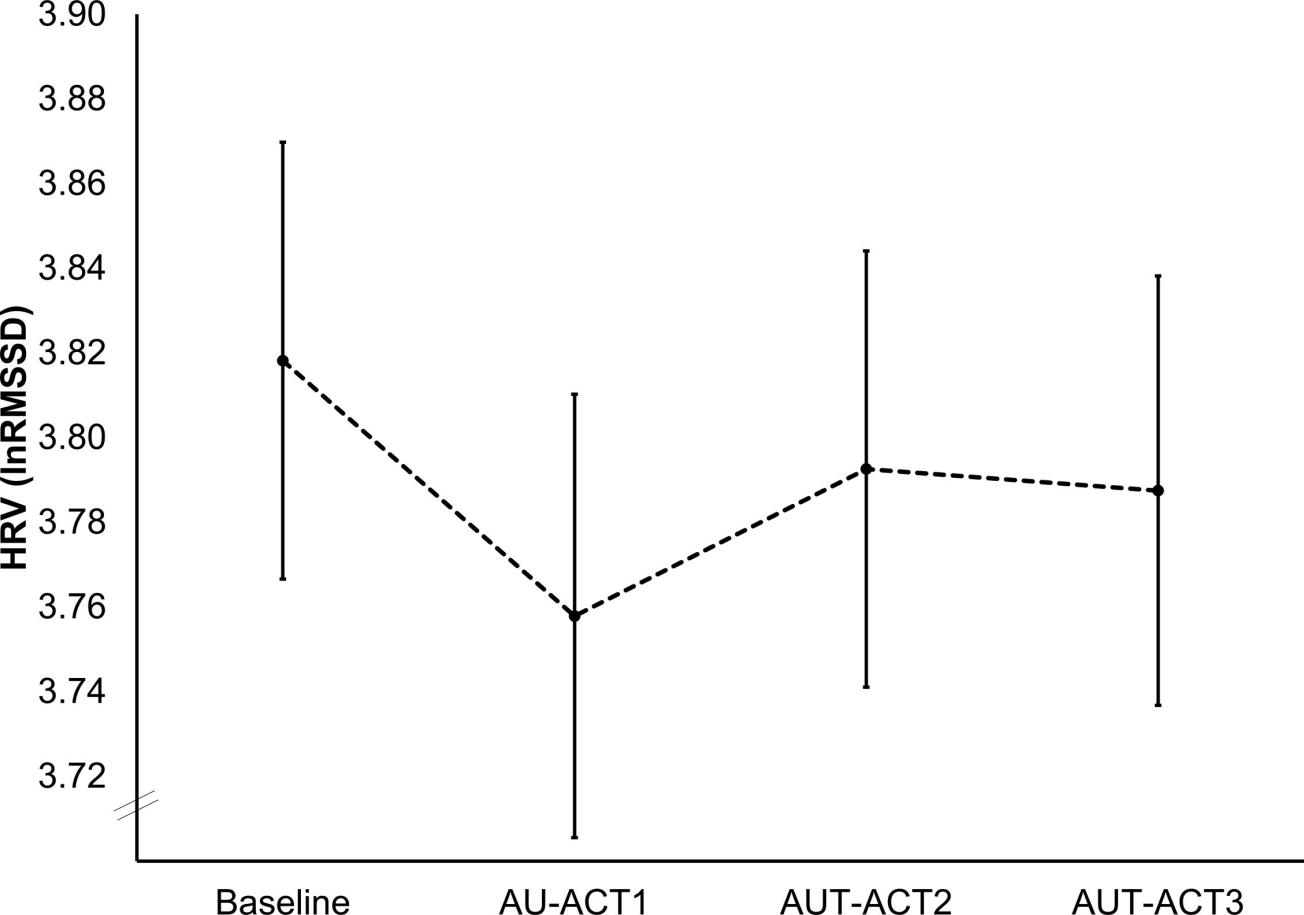
Mean HRV (lnRMSSD; error bars = *SE*) in the baseline and the three consecutive activation (ACT) intervals during the AU task (AU-ACT1, AU-ACT2, AU-ACT3).

The analysis slightly failed to reach a significant main effect for TIME (*F*(3,94) = 2.45, *p* = .069, *η*_*p*_^*2*^ = .03). As indicated in Fig 2, the strongest decrease of HRV was from baseline to the first interval of the creative ideation task.

### Association between task-related HRV (lnRMSSD) change and task performance (originality), and its moderation by the amount of regular exercise

The regression analysis was significant (*F*(3,93) = 4.50, *p =* .005, *R*^*2*^ = .127). As depicted in Table 1, the interaction effect of task-related HRV change by amount of physical exercise was significant (*β* = .34, *p* = .001), however the main effects of the amount of exercise and HRV change were non-significant. Please note that the results of the multiple regression analysis remained virtually the same when using pNN50 instead of RMSSD (*F*(3,93) = 2.98, *p =* .035, *R*^*2*^ = .09).

**Table 1:** Summary of the results of the multiple regression analysis for the prediction of task performance (originality) with task-related HRV (lnRMSSD) change, physical exercise, and the interaction term as predictors.

	*β*	*r*
Task-related HRV (lnRMSSD) change	.06 (.538)	.02 (.834)
Physical exercise	-.09 (.381)	-.13 (.194)
Task-related HRV (lnRMSSD) change x Physical exercise	.34 (.001)	.34 (.001)

*Note*. *p*-values in parenthesis. *r* represents the zero-order correlation and *β* the standardized Beta coefficient.

**Fig 3:**
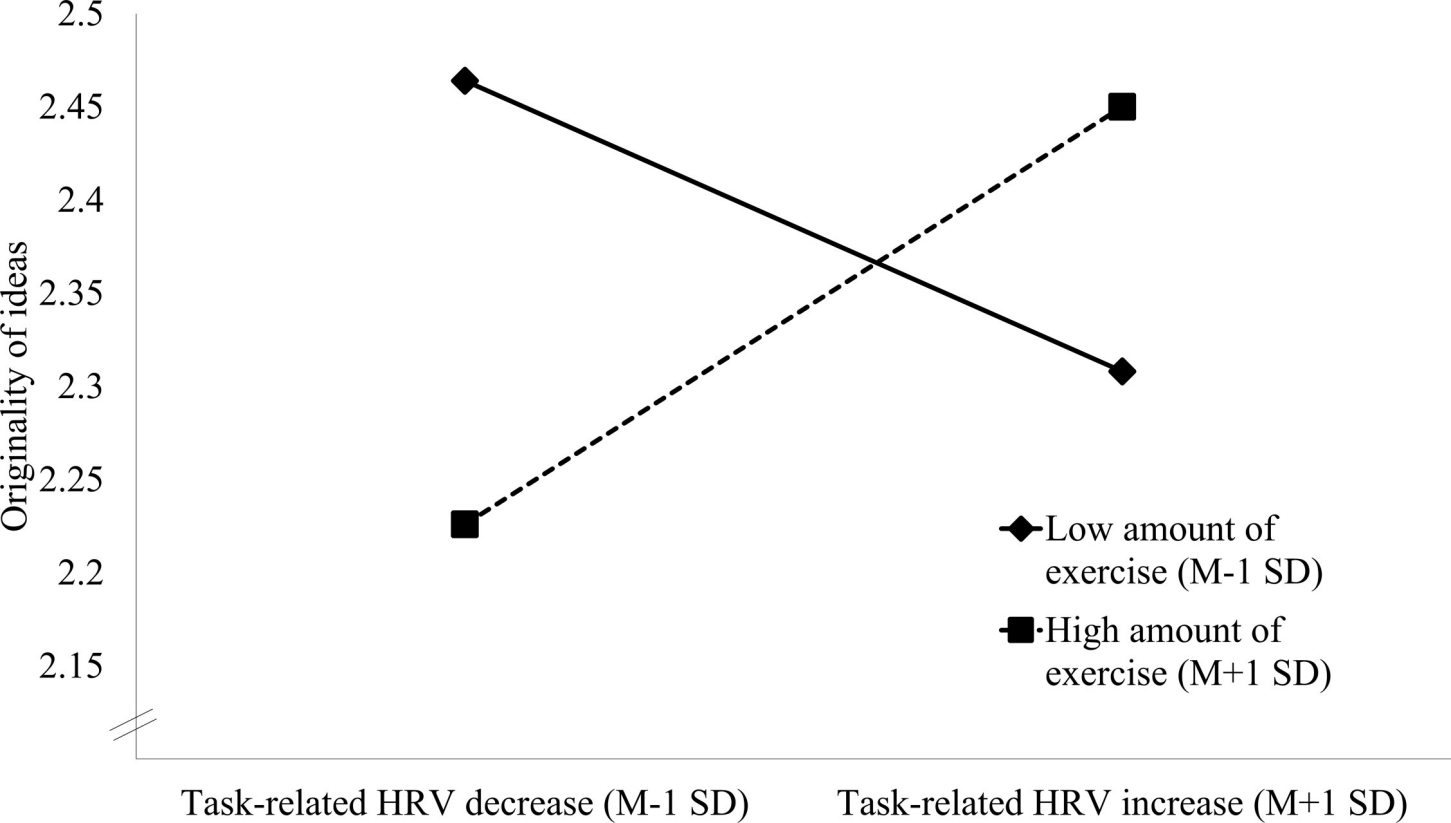
Interaction effect of task-related HRV change and the amount of regular physical exercise on task performance in terms of originality.

To further qualify the interaction effect in the whole sample, the regression analysis was re-run with the amount of regular exercise set as low (M—1 SD) and high (M + 1 SD; simple slopes approach), respectively. As illustrated in Fig 3, participants exercising more often performed better in the creative thinking task in terms of originality when they showed greater relative HRV increases during the challenge (*β* = .40, *t*(93) = -2.73, *p* = .008). In participants exercising less this association was reversed (*β* = -.28, *t*(93) = -2.13, *p* = .036).

The regression analysis with the additional predictor EDR frequency was again significant (*F*(4,92) = 4.20, *p =* .004, *R*^*2*^ = .155). The interaction effect of task-related HRV change by amount of physical exercise remained significant (*β* = .32, *p* = .001) and the EDR frequency was not significant (*β* = .13, *p* = .214).

The correlation between the originality score and the number of generated ideas during the six minutes activation period was *r* = .05 (*p* = .596), indicating that the effects of the regression analysis was also not influenced by the number of generated ideas.

## Discussion

The aim of this study was to examine the interplay of regular physical exercise and HRV reactivity during a challenging creative ideation task on task performance. Generally, HRV tended to decrease during the task, particularly at the beginning, which indicates the mobilization of mental effort and is characteristic of challenging performance tasks [87,88]. However, participants greatly varied in their cardiac response to the challenge. While some showed marked decreases of HRV, others exhibited smaller decreases or even increases of HRV during the task [15]. This variability in the cardiac responses was meaningfully related to task performance in terms of the originality of generated creative ideas. However, only when inter-individual differences in regular exercising were considered.

In people with a higher amount of regular exercise, greater HRV increase (i.e., vagal activation) during the creative ideation task was correlated with greater originality of the produced ideas, which is in accordance with some relevant research [40,41]. By contrast, in more sedentary people this association was reversed. Greater task-related HRV decrease (i.e., greater vagal withdrawal) was associated with better task performance in terms of originality, which is in line with other studies in the literature [35]. Importantly, regular exercising as such was not correlated with task performance, that is, on average, people exercising both more or less achieved similar levels of originality [52,89]. This null finding might take place because of study-design divergences. Contrary to former studies [51,53], we used a self-report measure of regular physical exercise [52]. Furthermore, while Gondola and Tuckman [53] reported increases in the fluency parameter of the AU task and Latorre Román et al. [51] found a positive effect for a composite score of multiple creative thinking tasks, we operationalized creativity by means of originality of ideas.

Nevertheless, the observed interaction effect suggests that greater originality in a creativity task is not only achieved by the investment of more mental effort [24,35], and more spontaneous and associative modes of thinking [32], but that both can lead to success, depending, among others, on the physical constitution of the person. This finding may in part explain the heterogeneity of previous results on associations between cardiac vagal control and creative performance [24,35,40].

Furthermore, the observed moderation effect at least in part supports assumptions of the cross-stressor adaptation theory [57], according to which changing cardiac response patterns to physical stressors by physical exercising may translate to altered modes of responding of regulation functions when confronted with psychological stressors [46,59,60]. People who generate more original ideas showed smaller vagal withdrawal (or even increases in heart rate variability) during task performance, when they were regularly exercising as compared to being more sedentary. This is in line with Park et al. [90], who reported task-related increases of HRV during a cognitive task in participants with higher flexible regulation functions (i.e., higher tonic vagal control) and decreases in participants with lower regulation functions (for short review see [15]).

The present findings need to be discussed in light of some limitations. First, the amount of regular physical exercise was only assessed by self-report and not by a behavioral index. Although the current findings need to be replicated with objective measures, similar self-reports of exercise have proven suitable to reveal reliable and meaningful associations with challenge-related cardiac responses [59]. Second, it might be proposed that HRV is influenced by verbal responses during the creative ideation task [91]. However, even if the vocalization of ideas has an impact on HRV, this cannot explain the reported associations between HRV and originality (for people exercising more or less, respectively), since the number of generated ideas (within the 6 minutes activation period) was virtually independent of task performance (originality). This is further in line with the result, that the respiration frequency (EDR) had no significant impact on the findings of this study. Third, although the eye closed baseline condition is the most frequently used approach in psychophysiological research [15], an additional baseline using an eyes open condition might have been valuable to evaluate the specificity of reported task-related HRV changes. Fourth, due to the cross-sectional, correlational nature of the present study, no cause-effect relationship can be deduced from the results.

Despite of these limitations, the findings of this research suggests that participants exercising more often in their daily life achieve higher originality with more flexible, associative, and spontaneous modes of thinking, while participants exhibiting a more sedentary lifestyle more strongly invest mental effort to reach the same quality level of produced ideas. Since solving open and ill-defined problems constitutes a daily mental challenge for many people such mechanisms might impose health risks and lower well-being in people with a sedentary lifestyle in the long run. Conversely, grounding on the cross-stressor adaptation theory the findings of the present research supports the beneficial effect of regular exercising on mental challenges in daily life [46,57].

### Conclusions

The current study provides evidence that creative performance depends on individual differences in task-related changes in vagal activity, when taking regular exercising into account. This supports the conclusions that firstly, regular physical exercising has an impact on creativity [51,53] and secondly, higher creativity can be achieved by different strategies such as spontaneous modes of thinking and mental effort [24,32].

## Supporting information

S1 Dataset (XLSX)

## References

[1] Thayer JF, Ahs F, Fredrikson M, Sollers JJ, Wager TD. A meta-analysis of heart rate variability and neuroimaging studies. Implications for heart rate variability as a marker of stress and health. Neuroscience and Biobehavioral Reviews. 2012; 36(2): 747–756. 10.1016/j.neubiorev.2011.11.009 22178086

[2] Porges SW. The polyvagal perspective. Biological Psychology. 2007; 74(2): 116–143. 10.1016/j.biopsycho.2006.06.009 17049418 PMC1868418

[3] Ingjaldsson JT, Laberg JC, Thayer JF. Reduced heart rate variability in chronic alcohol abuse. Relationship with negative mood, chronic thought suppression, and compulsive drinking. Biological Psychiatry. 2003; 54(12): 1427–1436. 10.1016/S0006-3223(02)01926-1 14675808

[4] Segerstrom SC, Nes LS. Heart rate variability reflects self-regulatory strength, effort, and fatigue. Psychological Science. 2007; 18(3): 275–281. 10.1111/j.1467-9280.2007.01888.x 17444926

[5] Thayer JF, Friedman BH. Stop that! Inhibition, sensitization, and their neurovisceral concomitants. Scand. J. Psychol. 2002; 43(2): 123–130. 10.1111/1467-9450.00277 12004949

[6] Duarte A, Soares PP, Pescatello L, Farinatti P. Aerobic training improves vagal reactivation regardless of resting vagal control. Medicine & Science in Sports & Exercise. 2015; 47(6): 1159–1167. 10.1249/MSS.0000000000000532 25259540

[7] Thayer JF, Lane RD. Claude Bernard and the heart-brain connection. Further elaboration of a model of neurovisceral integration. Neuroscience and Biobehavioral Reviews. 2009; 33(2): 81–88. 10.1016/j.neubiorev.2008.08.004 18771686

[8] Buch AN, Coote JH, Townend JN. Mortality, cardiac vagal control and physical training—what’s the link. Experimental Physiology. 2002; 87(4): 423–435. 10.1111/j.1469-445X.2002.tb00055.x 12392106

[9] Task Force Guidelines. Heart rate variability. Standards of measurement, physiological interpretation, and clinical use. Circulation. 1996; 93(5): 1043–1065. 10.1161/01.CIR.93.5.1043 8598068

[10] Shaffer F, Ginsberg JP. An overview of heart rate variability metrics and norms. Frontiers in Public Health. 2017; 5: 258 10.3389/fpubh.2017.00258 29034226 PMC5624990

[11] Hansen AL, Johnsen BH, Thayer JF. Vagal influence on working memory and attention. International Journal of Psychophysiology. 2003; 48(3): 263–274. 10.1016/S0167-8760(03)00073-4 12798986

[12] Johnsen BH, Thayer JF, Laberg JC, Wormnes B, Raadal M et al Attentional and physiological characteristics of patients with dental anxiety. Journal of Anxiety Disorders. 2003; 17(1): 75–87. 10.1016/S0887-6185(02)00178-0 12464290

[13] Butler EA, Wilhelm FH, Gross JJ. Respiratory sinus arrhythmia, emotion, and emotion regulation during social interaction. Psychophysiology. 2006; 43(6): 612–622. 10.1111/j.1469-8986.2006.00467.x 17076818

[14] Zahn D, Adams J, Krohn J, Wenzel M, Mann CG et al Heart rate variability and self-control-A meta-analysis. Biological Psychology. 2016; 115: 9–26. 10.1016/j.biopsycho.2015.12.007 26747415

[15] Laborde S, Mosley E, Thayer JF. Heart rate variability and cardiac vagal tone in psychophysiological research—recommendations for experiment planning, data analysis, and data reporting. Frontiers in Psychology. 2017; 8: 213 10.3389/fpsyg.2017.00213 28265249 PMC5316555

[16] Balzarotti S, Biassoni F, Colombo B, Ciceri MR. Cardiac vagal control as a marker of emotion regulation in healthy adults. A review. Biological Psychology. 2017; 130: 54–66. 10.1016/j.biopsycho.2017.10.008 29079304

[17] Graziano P, Derefinko K. Cardiac vagal control and children’s adaptive functioning. A meta-analysis. Biological Psychology. 2013; 94(1): 22–37. 10.1016/j.biopsycho.2013.04.011 23648264 PMC4074920

[18] Chrysikou EG, Weber MJ, Thompson-Schill SL. A matched filter hypothesis for cognitive control. Neuropsychologia. 2014; 62: 341–355. 10.1016/j.neuropsychologia.2013.10.021 24200920 PMC4010565

[19] Pinho AL, Ullen F, Castelo-Branco M, Fransson P, de Manzano O. Addressing a paradox: Dual strategies for creative performance in introspective and extrospective networks. Cerebral Cortex. 2016; 26(7): 3052–3063. 10.1093/cercor/bhv130 26088973

[20] Boutcher SH, Nugent FW, McLaren PF, Weltman AL. Heart period variability of trained and untrained men at rest and during mental challenge. Psychophysiology. 1998; 35(1): 16–22. 10.1017/S0048577298951435 9499702

[21] Overbeek TJM, van Boxtel A, Westerink JHDM. Respiratory sinus arrhythmia responses to cognitive tasks. Effects of task factors and RSA indices. Biological Psychology. 2014; 99: 1–14. 10.1016/j.biopsycho.2014.02.006 24561100

[22] Denson TF, Grisham JR, Moulds ML. Cognitive reappraisal increases heart rate variability in response to an anger provocation. Motiv Emot. 2011; 35(1): 14–22. 10.1007/s11031-011-9201-5

[23] Christou-Champi S, Farrow TFD, Webb TL. Automatic control of negative emotions: Evidence that structured practice increases the efficiency of emotion regulation. Cognition & Emotion. 2015; 29(2): 319–331. 10.1080/02699931.2014.901213 24678930 PMC4241596

[24] Silvia PJ, Beaty RE, Nusbaum EC, Eddington KM, Kwapil TR. Creative motivation. Creative achievement predicts cardiac autonomic markers of effort during divergent thinking. Biological Psychology. 2014; 102: 30–37. 10.1016/j.biopsycho.2014.07.010 25063471 PMC6211184

[25] Fink A, Weiss EM, Schwarzl U, Weber H, Assunção VL de et al Creative ways to well-being: Reappraisal inventiveness in the context of anger-evoking situations. Cognitive, Affective & Behavioral Neuroscience. 2017; 17(1): 94–105. 10.3758/s13415-016-0465-9 27683302 PMC5272882

[26] Perchtold CM, Papousek I, Koschutnig K, Rominger C, Weber H et al Affective creativity meets classic creativity in the scanner. Hum Brain Mapp. 2018; 39: 393–406. 10.1002/hbm.23851 29058352 PMC6866914

[27] Fink A, Perchtold C, Rominger C. 2018 Creativity and cognitive control in the cognitive and affective domains In: Jung RE, Vartanian O, editors. The Cambridge handbook of the neuroscience of creativity. Cambridge: Cambridge University Press pp. 318–332.

[28] Rominger C, Papousek I, Weiss EM, Schulter G, Perchtold CM et al Creative thinking in an emotional context: Specific relevance of executive control of emotion-laden representations in the inventiveness in generating alternative appraisals of negative events. Creativity Res. J. 2018; 30(3): 256–265. 10.1080/10400419.2018.1488196

[29] Beaty RE, Benedek M, Silvia PJ, Schacter DL. Creative cognition and brain network dynamics. Trends in Cognitive Sciences. 2016; 20(2): 87–95. 10.1016/j.tics.2015.10.004 26553223 PMC4724474

[30] Rominger C, Fink A, Weiss EM, Bosch J, Papousek I. Allusive thinking (remote associations) and auditory top-down inhibition skills differentially predict creativity and positive schizotypy. Cognitive Neuropsychiatry. 2017; 22(2): 108–121. 10.1080/13546805.2016.1278361 28081654

[31] Sowden PT, Pringle A, Gabora L. The shifting sands of creative thinking. Connections to dual-process theory. Thinking and Reasoning. 2015; 21(1): 40–60. 10.1080/13546783.2014.885464

[32] Benedek M, Jauk E. 2018 Spontaneous and controlled processes in creative cognition In: Fox KCR, Christoff K, editors. The Oxford handbook of spontaneous thought. Mind-wandering, creativity, and dreaming. New York, NY: Oxford University Press.

[33] Mok LW. The interplay between spontaneous and controlled processing in creative cognition. Frontiers in Human Neuroscience. 2014; 8: 663 10.3389/fnhum.2014.00663 25221497 PMC4147391

[34] Allen AP, Thomas KE. A dual process account of creative thinking. Creativity Research Journal. 2011; 23(2): 109–118. 10.1080/10400419.2011.571183

[35] Loudon GH, Deininger GM. The physiological response during divergent thinking. JBBS. 2016; 06(01): 28–37. 10.4236/jbbs.2016.61004

[36] Jauk EV, Benedek M, Neubauer AC. The road to creative achievement: A latent variable model of ability and personality predictors. European Journal of Personality. 2014; 28(1): 95–105. 10.1002/per.1941 24532953 PMC3923982

[37] Nusbaum EC, Silvia PJ. Are intelligence and creativity really so different? Fluid intelligence, executive processes, and strategy use in divergent thinking. Intelligence. 2011; 39(1): 36–45. 10.1016/j.intell.2010.11.002

[38] Runco MA, Chand I. Cognition and creativity. Educ Psychol Rev. 1995; 7(3): 243–267. 10.1007/BF02213373

[39] Martindale C. 1999 Biological basis of creativity In: Sternberg RJ, editor. Handbook of creativity. Cambridge: Cambridge University Press pp. 137–152.

[40] Bowers KS, Keeling KR. Heart-rate variability in creative functioning. Psychological Reports. 1971; 29(1): 160–162. 10.2466/pr0.1971.29.1.160 4937323

[41] Blatt SJ. Patterns of cardiac arousal during complex mental activity. The Journal of Abnormal and Social Psychology. 1961; 63(2): 272–282. 10.1037/h0044753 13869907

[42] Mednick SA. The associative basis of the creative process. Psychological Review. 1962; 69(3): 220–232. 10.1037/h0048850 14472013

[43] Ellamil M, Dobson C, Beeman M, Christoff K. Evaluative and generative modes of thought during the creative process. NeuroImage. 2012; 59(2): 1783–1794. 10.1016/j.neuroimage.2011.08.008 21854855

[44] Rominger C, Papousek I, Perchtold CM, Weber B, Weiss EM et al The creative brain in the figural domain. Distinct patterns of EEG alpha power during idea generation and idea elaboration. Neuropsychologia. 2018; 118(Part A): 13–19. 10.1016/j.neuropsychologia.2018.02.013 29452125

[45] Finke RA, Ward TB, Smith SM. 1996 Creative cognition Theory, research, and applications. Cambridge, Mass: MIT Press. 239 p.

[46] Forcier K, Stroud LR, Papandonatos GD, Hitsman B, Reiches M et al Links between physical fitness and cardiovascular reactivity and recovery to psychological stressors. A meta-analysis. Health Psychology. 2006; 25(6): 723–739. 10.1037/0278-6133.25.6.723 17100501

[47] Colcombe S, Kramer AF. Fitness effects on the cognitive function of older adults. A meta-analytic study. Psychol Sci. 2003; 14(2): 125–130. 10.1111/1467-9280.t01-1-01430 12661673

[48] Etnier JL, Salazar W, Landers DM, Petruzzello SJ, Han M et al The influence of physical fitness and exercise upon cognitive functioning. A meta-analysis. Journal of Sport and Exercise Psychology. 1997; 19(3): 249–277. 10.1123/jsep.19.3.249

[49] Young J, Angevaren M, Rusted J, Tabet N. Aerobic exercise to improve cognitive function in older people without known cognitive impairment. The Cochrane Database of Systematic Reviews. 2015; (4): CD005381 10.1002/14651858.CD005381.pub4 25900537 PMC10554155

[50] Albinet CT, Abou-Dest A, André N, Audiffren M. Executive functions improvement following a 5-month aquaerobics program in older adults. Role of cardiac vagal control in inhibition performance. Biological Psychology. 2016; 115: 69–77. 10.1016/j.biopsycho.2016.01.010 26812613

[51] Latorre Román PÁ, Pinillos FG, Pantoja Vallejo A, Berrios Aguayo B. Creativity and physical fitness in primary school-aged children. Pediatrics International. 2017; 59(11): 1194–1199. 10.1111/ped.13391 28802081

[52] Cavallera GM, Boari G, Labbrozzi D, Bello ED. Morningness-eveningness personality and creative thinking among young people who play recreational sport. Soc Behav Personal. 2011; 39(4): 503–518. 10.2224/sbp.2011.39.4.503

[53] Gondola JC, Tuckman BW. Effects of a systematic program of exercise on selected measures of creativity. Perceptual and Motor Skills. 1985; 60(1): 53–54. 10.2466/pms.1985.60.1.53 3982945

[54] Latorre Román PÁ, Pantoja Vallejo A, Berrios Aguayo B. Acute Aerobic Exercise Enhances Students’ Creativity. Creativity Research Journal. 2018; 30(3): 310–315. 10.1080/10400419.2018.1488198

[55] Steinberg H, Sykes EA, Moss T, Lowery S, LeBoutillier N et al Exercise enhances creativity independently of mood. British Journal of Sports Medicine. 1997; 31(3): 240–245. 10.1136/bjsm.31.3.240 9298561 PMC1332529

[56] Blanchette DM, Ramocki SP, O’del JN, Casey MS. Aerobic exercise and creative potential. Immediate and residual effects. Creativity Research Journal. 2005; 17(2–3): 257–264. 10.1080/10400419.2005.9651483

[57] Sothmann MS, Buckworth J, Claytor RP, Cox RHON, White-Welkley JE et al Exercise training and the cross-stressor adaptation hypothesis. Exercise and Sport Sciences Reviews. 1996; 24(1). 8744253

[58] Spalding TW, Lyon LA, Steel DH, Hatfield BD. Aerobic exercise training and cardiovascular reactivity to psychological stress in sedentary young normotensive men and women. Psychophysiology. 2004; 41(4): 552–562. 10.1111/j.1469-8986.2004.00184.x 15189478

[59] Lackner HK, Weiss EM, Hofer E, Rössler A, Fink A et al Transient cardiac responses to witnessing horrible events in young adult female exercisers and non-exercisers. Psychology of Sport and Exercise. 2016; 22: 312–320. 10.1016/j.psychsport.2015.09.006

[60] Luque-Casado A, Zabala M, Morales E, Mateo-March M, Sanabria D. Cognitive performance and heart rate variability: The influence of fitness level. PLoS ONE. 2013; 8(2): e56935 10.1371/journal.pone.0056935 23437276 PMC3577676

[61] Faul F, Erdfelder E, Buchner A, Lang A-G. Statistical power analyses using G*Power 3.1: Tests for correlation and regression analyses. Behav Res. 2009; 41(4): 1149–1160. 10.3758/BRM.41.4.1149 19897823

[62] Steingrüber H-J. 2010 Hand-Dominanz-Test H-D-T. Göttingen: Hogrefe.

[63] Frey I, Berg A, Grathwohl D, Keul J. Freiburger Fragebogen zur körperlichen Aktivität-Entwicklung, Prüfung und Anwendung. Soz Präventivmed. 1999; 44(2): 55–64. 10.1007/BF01667127 10407953

[64] Rogge A-K, Röder B, Zech A, Nagel V, Hollander K et al Balance training improves memory and spatial cognition in healthy adults. Scientific Reports. 2017; 7(1): 5661 10.1038/s41598-017-06071-9 28720898 PMC5515881

[65] Kleemeyer MM, Kühn S, Prindle J, Bodammer NC, Brechtel L et al Changes in fitness are associated with changes in hippocampal microstructure and hippocampal volume among older adults. NeuroImage. 2016; 131: 155–161. 10.1016/j.neuroimage.2015.11.026 26584869

[66] Ainsworth BE, Haskell WL, Whitt MC, Irwin ML, Swartz AM et al Compendium of physical activities. An update of activity codes and MET intensities. Medicine & Science in Sports & Exercise. 2000; 32(9 SUPPL): S498–S504. 10993420 10.1097/00005768-200009001-00009

[67] Caspersen CJ, Powell KE, Christenson GM. Physical activity, exercise, and physical fitness: definitions and distinctions for health-related research. Public Health Reports. 1985; 100(2): 126–131. 3920711 PMC1424733

[68] Chang Y-K, Chi L, Etnier JL, Wang C-C, Chu C-H et al Effect of acute aerobic exercise on cognitive performance. Role of cardiovascular fitness. Psychology of Sport and Exercise. 2014; 15(5): 464–470. 10.1016/j.psychsport.2014.04.007

[69] Guilford JP. 1967 The nature of human intelligence. New York: McGraw-Hill. [XII], 537 p.

[70] Fink A, Benedek M, Grabner RH, Staudt B, Neubauer AC. Creativity meets neuroscience: Experimental tasks for the neuroscientific study of creative thinking. Methods. 2007; 42(1): 68–76. 10.1016/j.ymeth.2006.12.001 17434417

[71] Benedek M, Jauk E, Fink A, Koschutnig K, Reishofer G et al To create or to recall? Neural mechanisms underlying the generation of creative new ideas. Neuroimage. 2014; 88: 125–133. 10.1016/j.neuroimage.2013.11.021 24269573 PMC3991848

[72] Finke RA. 1996 Imagery, creativity, and emergent structure. 13 p. 10.1006/ccog.1996.0024 8906409

[73] Benedek M, Schües T, Beaty RE, Jauk E, Koschutnig K et al To create or to recall original ideas. Brain processes associated with the imagination of novel object uses. Cortex. 2018; 99: 93–102. 10.1016/j.cortex.2017.10.024 29197665 PMC5796649

[74] Jauk EV, Benedek M, Neubauer AC. Tackling creativity at its roots: Evidence for different patterns of EEG alpha activity related to convergent and divergent modes of task processing. International Journal of Psychophysiology. 2012; 84(2): 219–225. 10.1016/j.ijpsycho.2012.02.012 22390860 PMC3343259

[75] Fink A, Rominger C, Benedek M, Perchtold CM, Papousek I et al EEG alpha activity during imagining creative moves in soccer decision-making situations. Neuropsychologia. 2018; (114): 118–124. 10.1016/j.neuropsychologia.2018.04.025 29702162

[76] Amabile TM. Social psychology of creativity: A consensual assessment technique. Journal of Personality and Social Psychology. 1982; 43(5): 997–1013. 10.1037/0022-3514.43.5.997

[77] Shaffer F, McCraty R, Zerr CL. A healthy heart is not a metronome: An integrative review of the heart’s anatomy and heart rate variability. Frontiers in Psychology. 2014; 5: 1040 10.3389/fpsyg.2014.01040 25324790 PMC4179748

[78] Munoz ML, van Roon A, Riese H, Thio C, Oostenbroek E et al Validity of (Ultra-)Short Recordings for Heart Rate Variability Measurements. PLoS ONE. 2015; 10(9): e0138921 10.1371/journal.pone.0138921 26414314 PMC4586373

[79] Luque-Casado A, Perales JC, Cárdenas D, Sanabria D. Heart rate variability and cognitive processing: The autonomic response to task demands. Biological Psychology. 2016; 113: 83–90. 10.1016/j.biopsycho.2015.11.013 26638762

[80] Gerteis AKS, Schwerdtfeger AR. When rumination counts. Perceived social support and heart rate variability in daily life. Psychophysiology. 2016; 53(7): 1034–1043. 10.1111/psyp.12652 27137911

[81] Schwerdtfeger AR, Gerteis AKS. The manifold effects of positive affect on heart rate variability in everyday life. Distinguishing within-person and between-person associations. Health Psychology. 2014; 33(9): 1065–1073. 10.1037/hea0000079 24707841

[82] Schwerdtfeger AR, Dick K. Episodes of momentary resilience in daily life are associated with HRV reductions to stressful operations in firefighters: an ambulatory assessment approach using bayesian multilevel modeling. 2018; 47(4): 1–10. 10.1080/17439760.2018.1497689

[83] Vasey MW, Thayer JF. The continuing problem of false positives in repeated measures ANOVA in psychophysiology: A multivariate solution. Psychophysiology. 1987; 24(4): 479–486. 3615759 10.1111/j.1469-8986.1987.tb00324.x

[84] Llabre MM, Spitzer SB, Saab PG, Ironson GH, Schneiderman N. The reliability and specificity of delta versus residualized change as measures of cardiovascular reactivity to behavioral challenges. Psychophysiology. 1991; 28(6): 701–711. 10.1111/j.1469-8986.1991.tb01017.x 1816598

[85] Rominger C, Papousek I, Perchtold CM, Benedek M, Weiss EM et al Creativity is associated with a characteristic U-shaped function of alpha power changes accompanied by an early increase in functional coupling. Cognitive, Affective, & Behavioral Neuroscience. in press. 10.3758/s13415-019-00699-y 30756348 PMC6711878

[86] Schwerdtfeger AR, Friedrich-Mai P. Social interaction moderates the relationship between depressive mood and heart rate variability. Evidence from an ambulatory monitoring study. Health Psychology. 2009; 28(4): 501–509. 10.1037/a0014664 19594275

[87] Wood R, Maraj B, Lee MC, Reyes R. Short-term heart rate variability during a cognitive challenge in young and older adults. Age and Ageing. 2002; 31(2): 131–135. 10.1093/ageing/31.2.131 11937476

[88] Fairclough SH, Mulder LJM. 2012 Psychophysiological processes of mental effort investment In: Wright RA, Gendolla GHE, editors. How motivation affects cardiovascular response. Mechanisms and applications. Washington, D.C: American Psychological Association pp. 61–76.

[89] Etnier JL, Nowell PM, Landers DM, Sibley BA. A meta-regression to examine the relationship between aerobic fitness and cognitive performance. Brain Research Reviews. 2006; 52(1): 119–130. 10.1016/j.brainresrev.2006.01.002 16490256

[90] Park G, Vasey MW, van Bavel JJ, Thayer JF. When tonic cardiac vagal tone predicts changes in phasic vagal tone: the role of fear and perceptual load. Psychophysiology. 2014; 51(5): 419–426. 10.1111/psyp.12186 24571084

[91] Grossman P, Taylor EW. Toward understanding respiratory sinus arrhythmia. Relations to cardiac vagal tone, evolution and biobehavioral functions. Biological Psychology. 2007; 74(2): 263–285. 10.1016/j.biopsycho.2005.11.014 17081672

